# Secure TPMS Data Transmission in Real-Time IoV Environments: A Study on 5G and LoRa Networks

**DOI:** 10.3390/s26020358

**Published:** 2026-01-06

**Authors:** D. K. Niranjan, Muthuraman Supriya, Walter Tiberti

**Affiliations:** 1 Department of Computer Science and Engineering, Amrita School of Computing, Amrita Vishwa Vidyapeetham, Bengaluru 560035, India; 2 Department DISIM and Centre Ex-EMERGE, University of L’Aquila, Via Vetoio 1, 67100 L’Aquila, Italy; walter.tiberti@univaq.it

**Keywords:** internet of vehicle, vehicle to vehicle, tire pressure monitoring system, air pressure, security, Raspberry Pi, sensor monitoring, 5G, LoRa

## Abstract

The advancement of Automotive Industry 4.0 has promoted the development of Vehicle to Vehicle (V2V) and Internet of Vehicles (IoV) communication, which marks the new era for intelligent, connected and automated transportation. Despite the benefits of this metamorphosis in terms of effectiveness and convenience, new obstacles to safety, inter-connectivity, and cybersecurity emerge. The tire pressure monitoring system (TPMS) is one prominent feature that senses tire pressure, which is closely related to vehicle stability, braking performance and fuel efficiency. However, the majority of TPMSs currently in use are based on the use of insecure and proprietary wireless communication links that can be breached by attackers so as to interfere with not only tire pressure readings but also sensor data manipulation. For this purpose, we design a secure TPMS architecture suitable for real-time IoV sensing. The framework is experimentally implemented using a Raspberry Pi 3B+ (Raspberry Pi Ltd., Cambridge, UK) as an independent autonomous control unit (ACU), interfaced with vehicular pressure sensors and a LoRa SX1278 (Semtech Corporation, Camarillo, CA, USA) module to support low-power, long-range communication. The gathered sensor data are encrypted, their integrity checked, source authenticated by lightweight cryptographic algorithms and sent to a secure server locally. To validate this approach, we show a three-node exhibition where Node A (raw data and tampered copy), B (unprotected copy) and C (secure auditor equipped with alerting of tampering and weekly rotation of the ID) realize detection of physical level threats at top speeds. The validated datasets are further enriched in a MATLAB R2024a simulator by replicating the data of one vehicle by 100 virtual vehicles communicating using over 5G, LoRaWAN and LoRa P2P as communication protocols under urban, rural and hill-station scenarios. The presented statistics show that, despite 5G ultra-low latency, LoRa P2P consistently provides better reliability and energy efficiency and is more resistant to attacks in the presence of various terrains. Considering the lack of private vehicular 5G infrastructure and the regulatory restrictions, this work simulated and evaluated the performance of 5G communication, while LoRa-based communication was experimentally validated with a hardware prototype. The results underline the trade-offs among LoRa P2P and an infrastructure-based uplink 5G mode, when under some specific simulation conditions, as opposed to claiming superiority over all 5G modes. In conclusion, the presented Raspberry Pi–MATLAB hybrid solution proves to be an effective and scalable approach to secure TPMS in IoV settings, intersecting real-world sensing with large-scale network simulation, thus enabling safer and smarter next-generation vehicular systems.

## 1. Introduction

The global automotive sector is in the midst of a wholesale transformation driven by digitization, connectivity and automation. In the last two decades, vehicles have become more than just mechanical devices that carry people or goods; they are now intelligent machines with cyber-physical systems and are able to perceive, decide and communicate in real time. This revolution, or what we refer to as Automotive Industry 4.0, brings the intersection of embedded electronics, wireless networking, cloud computing and artificial intelligence into every vehicle manufactured today. Whereas it used to be a self-contained mechanical apparatus, it now acts as a node in a sprawling network of connected data.

The market growth of the global Internet of Vehicles (IoV) has shown significant expansion. The market is estimated to expand from USD 173.90 billion in 2024 to USD 972.59 billion by 2032, at a Compound Annual Growth Rate (CAGR) of 24% throughout the forecast period. Asia Pacific occupied the majority of the market share, with 38.95% [1,2]. At the core of this development is the IoV, which consists of three primary network domains:

(1) A vehicular mobile internet that enables vehicles to communicate with cloud infrastructure, roadside units, and mobility management services.
(2) An intra-vehicular network that connects sensors, ECUs, and actuators inside each vehicle.
(3) An inter-vehicle network that accommodates direct V2V communication for cooperative sensing and collision avoidance.

These layers combine to create a flexible structure for self-driving, predictive diagnostics, over-the-air updates and fleet management. This architecture is evolving with the car as an element of the universal web—along with volumes of terabytes/day being produced and consumed [1,3].

The never-before-seen digital convergence also brings about deep challenges. Each new sensor, Electronic Control Unit (ECU) and communication interface is a potential open door for a hacker or offensive code. A single compromised node may disseminate the false information throughout the entire network, leading to wrong control decisions or privacy loss. Vehicle safety today, in other words, has as much to do with the integrity of digital data as it does with the soundness of its metal. Growing reliance on control by software (for example, key safety functions such as braking, steering and stability management) results in a possible accident or malfeasance with respect to data communication, leading directly to hazardous situations [4,5].

The complexity is evident in the modern vehicle design. A modern vehicle includes, on average, 70 to 150 ECUs that are used to manage a particular system (powertrain, infotainment, climate control, or driving assistance). These are usually software-enabled controllers and have separate firmware, microcontroller and communication interfaces. This modular architecture is solid for flexibility and scalability, but it also increases the possible attack surface. A dedicated protocol is normally used for each of the ECUs—such as CAN, LIN, FlexRay, or automotive Ethernet—however, these networks are only lightweight and isolated. If an attacker can compromise one ECU, for example, through infotainment or telematics, they might be able to move across internal buses towards safety-critical ECUs such as steering and brakes [6,7].

This heterogeneity and distribution of control and communication provide fertile ground for manipulation, although it is often necessary for functionality. Several high-profile automotive cyber-attacks have resulted in wireless ECU remote exploitation; hence, the motivation for common encryption, intrusion detection and authenticated inter-controller communication [8].

In such an interlinked control ecosystem, the state of health of the tire remains a crucial safety parameter. The tires are the only thing between the vehicle and the road, so they directly impact traction, braking effectiveness and stability. Especially in emergency stopping applications, any variation can increase stopping distance and risk of skidding or a possible accident. Research indicates that a lot of tire-related accidents result from communication mistakes or pressure monitoring system delays, and not mechanical failure. False or manipulated sensor data that is fed to the vehicle control algorithms can result in dangerous reactions—especially in semi-autonomous cars, where sensor fusion is a condition of judgment [9].

Traditional TPMS sends data value from the sensors to the ECU or dashboard in a low-power RF module. The problem is that those transmissions are rarely encrypted and rarely authenticated, making it easy for an adversary to both eavesdrop (passive) or tamper with (active) packets using a cheap receiver. Such an alteration could result in false alarms, covering up important errors, or revealing individual sensor constants that can be related to particular vehicles. In interconnected IoV networks, where TPMS data are distributed to mobile applications or cloud dashboards, these vulnerabilities may become vehicle-centric security threats. As a consequence, TPMS is no longer relegated to being just a maintenance sub-system but rather an attack surface within the larger realm of IoV [7,10].

While TPMS information may seem light and not critical, untrusted access to this telemetry can support vehicle profiling, driving behavior inference and development of targeted replay or spoofing attacks. In today’s systems, TPMS data can be received and processed by mobile applications, cloud dashboards, or driver assistance software, so confidentiality is an important need in addition to integrity and authentication. Thus, the encryption is required to protect from passive eavesdropping, context information and building of malicious injection patterns.

Extending our prior physical-access security architecture, this proposed work assures the integrity of inter-vehicle communication and vehicle-embedded controllers via USB-based authorizations. Only the authenticated hardware tokens could act as triggers or modifiers for essential vehicle functions, which served as a secure foundation to protect the physical link from unauthorized access. This article advances this notion into a full-fledged secure communication infrastructure for TPMS and inter-vehicle telemetry, and brings attention to encrypted data exchange semantics in conjunction with integrity verification considerations, as well as scalability analysis under diverse communication media.

In this paper, a secure edge node for IoV in the form of Raspberry Pi 3B+ combined with LoRaSX1278 transceivers is realized to gather and send tire-pressure/temperature information. Every transmission frame is encrypted by AES-128 and is authenticated, making the MAC proof against eavesdropping or tamper attacks. Real-time encrypted data collected from the physical test-bed is then imported into a MATLAB-based V2X simulation architecture representing a single vehicle node to propagate it to one hundred virtual vehicles. The simulator models urban, rural, and hilly terrains, thus enabling the comparison of 5G with LoRaWAN and LoRa Peer-to-Peer (P2P) technologies while simulating the same type of attack and load [11,12].

Through the high integration of low-power telemetry, the validation of edge communication security through encryption and by reusing the access control enforcement employed, this study demonstrates a unified and scalable solution for securing tire pressure data and autonomous control units for Connected and Autonomous Vehicles (CAVs). This work exhibits that, by strengthening a single small system such as TPMS, we can substantially advance toward the ultimate goal that is resilient, secure and trustworthy vehicle-to-vehicle communication in the smart IoV world.

It is also important to mention that this work is not intended to reproduce the mechanical limitations or packaging constraints of commercial wheel-mounted TPMS devices. Instead, the platform introduced here is a research-based secure TPMS telemetry framework that can be used to study end-to-end encryption and authentication, as well as the resilience of attacks in IoV communication pipelines. This paper does not concern product-level mechanical integration, but rather focuses on data security, communication reliability and large-scale IoV behavior.

Unlike in our past works, this work centers on ensuring physical-layer security for end-to-end encryption-based TPMS telemetry data. It involves AES-HMAC encryption on the sensor and edge layers and also includes a 5G, LoRaWAN, and LoRa P2P IoV simulation environment.

The main contributions of this article are as follows:

- We propose a unified secure TPMS communication framework that integrates real-time tire-pressure data acquisition with encrypted LoRa-based transmission for the Internet of Vehicles. The system uses a Raspberry Pi 3B+ and LoRa SX1278 modules implementing AES-128 encryption and HMAC authentication to ensure confidentiality, integrity, and authenticity of tire-pressure telemetry against interception, replay, and tampering attacks.
- Extending our prior work, a physical access security architecture is proposed where the integrity of inter-vehicle communication and vehicle-embedded controllers via USB-based authorizations is assured. USB-based physical access authorization is included to secure wireless communication between vehicular units. The proposed framework unifies physical and cyber-layer protection, ensuring that both autonomous control units and inter-vehicle data links remain secure from unauthorized access and physical tampering within the IoV ecosystem [13].
- A cross-domain experimental and simulation-based evaluation is performed by integrating encrypted real-time TPMS data into a MATLAB V2X simulation that scales to one hundred virtual vehicles operating in urban, rural, and hill environments. Comparative analysis across 5G, LoRaWAN, and LoRa P2P networks demonstrates that secure LoRa P2P achieves the highest packet-delivery reliability, energy efficiency, and resilience under attack conditions [14,15].

The rest of this article is structured as follows. Section 2 reviews the related work. Section 3 describes the proposed model’s components in detail. Section 4 outlines the experimental setup and the evaluation of experimental results. Lastly, Section 5 provides the main conclusions and directions for future research.

## 2. Related Works

This paper discusses the related work on TPMS security, in-vehicle control vulnerabilities and communication technologies that are applied in IoV. Although a large amount of effort has been placed into the development of safety and connectivity methods for vehicles, few works have addressed low-cost and lightweight security mechanisms capable of protecting both sensing and communication layers.

### 2.1. TPMS Security and Data Integrity

Modern tire Pressure Monitoring Systems (TPMSs) have evolved from basic RF transmitters to intelligent sensor nodes that continuously measure tire pressure, temperature, and health parameters. However, since most commercial TPMS units transmit unencrypted frames, data integrity and authenticity remain major concerns. Research has shown that attackers within short range can intercept, replay, or inject falsified TPMS packets, leading to false alerts or masked tire failures [16,17].

To counter these issues, several studies propose lightweight cryptographic frameworks, such as AES-128, PRESENT, and HMAC-SHA256, to ensure data confidentiality and integrity while maintaining low power consumption. Other solutions deploy dynamic identifier randomization to address tracking and replay attacks [18]. Even so, end-to-end encryption, per-node authentication, and physical access protection (i.e., USB-based access control) are not commonly used in real-world deployments. The integrity in very low-power embedded environments also continues to be a challenge due to the computational load of cryptographic operations and their implications on battery life [19,20].

For further development, TPMS nodes in the next generation need to be designed with a security-aware embedded system so that each sensor node is capable of achieving a tradeoff between energy saving, message authentication, encryption and breakable detection for safe operation in the connected car system.

### 2.2. Vulnerabilities in Electronic Control Units (ECUs)

The Electronic Control Unit (ECU) is the central node for decisions that gather sensor data, including TPMS information and sends it through in-vehicle networks like CAN or FlexRay bus. Many studies have disclosed threats due to insecure Controller Area Network (CAN) message transmissions, lack of security mechanisms such as message authentication and the existence of diagnostic interfaces, which could be used for performing malicious reprogramming or injecting messages [21].

Attackers who gain access to the second generation of on-board self-diagnostic (OBD-II) or service ports can inject false CAN frames, disable safety functions, or manipulate tire data from TPMS sensors. Additionally, ECUs often trust incoming sensor data without source validation, allowing man-in-the-middle or replay attacks to propagate erroneous information. Recent research explores ECU-level defenses, including hardware-based roots of trust, firmware attestation, and intrusion detection modules (IDS) that monitor message patterns for abnormal behavior [22].

However, integrating these countermeasures increases hardware cost and system complexity, limiting their suitability for low-cost TPMSs. Hence, there is a growing need for edge-secured embedded nodes that can locally verify and encrypt data before forwarding it to the ECU, ensuring that only authenticated and tamper-proof information is processed.

### 2.3. IoV Communication Technologies: 5G, LoRaWAN, and LoRa P2P

Vehicle-to-Everything (V2X) and Internet-of-Vehicles (IoV) architectures increasingly rely on advanced wireless communication to achieve safety, scalability, and real-time data exchange. Among the various technologies explored for vehicular telemetry, 5G, LoRaWAN, and LoRa P2P are considered the most promising for TPMS and similar automotive applications.

- Fifth-generation (5G) New Radio offers ultra-low latency and high throughput, making it suitable for autonomous driving, sensor fusion, and cooperative awareness. It supports network slicing and end-to-end QoS control but depends heavily on operator infrastructure, incurs higher deployment cost, and consumes significant energy, limiting its suitability for small embedded nodes such as TPMS transmitters [23].
- LoRaWAN: LoRaWAN is able to offer long-range coverage and its network structure compounding a centralized star-of-stars topology, which is from end-devices connecting gateways to forwarding data to network servers. Device authentication and message integrity are guaranteed through AES-128 CMAC-based session keys, but the protocol brings in gateway availability overheads, join latency and cloud processing delays [24]. It is suitable for fleet tracking; however, because it is hosted in a data centre, the latency can be detrimental in time-critical automotive scenarios.
- LoRa P2P: LoRa Point-to-Point utilizes end device to end device reference without any need for gateways or network servers. This infrastructureless approach is of lower latency and has no attack surface than those generated by centralized approaches, which, in turn, will help push smart devices to have distinct support for evolutionary utility IoT systems [25]. It can be directly integrated into the local determent AES encryption, as well as HMAC security checking, thus enabling TPMS nodes and ECUs to communicate securely in real time and efficiently with low-power consumption.
  Recent comparative studies indicate that LoRa P2P provides high packet delivery ratios in both urban and rural mobility, with more energy-efficient operation towards cellular technologies while supporting lower bandwidth [14]. Therefore, LoRa P2P is a promising solution for secure, low-power and infrastructure-less vehicular telemetry.

### 2.4. Comparison with Current Secure TPMS and IoV Paradigms

Table 1 summarizes the existing works on TPMS security and TPMS/IoV networking mechanisms. The comparison of existing solutions is centered around the communication technologies used, their security schemes and the main limitations. Summarizing this survey, it could be concluded that the majority of the related work concentrates on high-level network security, and do not support embedded hardware authentication, nor provide end-to-end secure protection at sensor and edge-end for TPMS data.

As seen in Table 1, traditional TPMS and IoV solutions use either unsecured communication links via RF transmission in a static handshake or a gateway-based approach. This makes them vulnerable to spoofing attacks, replay attacks, and tampering attacks. Even though current studies have tackled network-level security concerns or edge-level approaches centered on ECUs, they have never considered the trust that exists in sensor-to-edge communication. The proposed system adds edge-level encryption and authentication along with physical access control and related estimation methods capable of scaling from sensor nodes all the way up to the cloud.

As can be noted from Table 1, in contrast to conventional TPMS and IoV solutions, which practice unencrypted RF communication or run over static handshakes or gateway-based architectures, the result is prone to spoofing, replay and tampering attacks. Though some works discuss network-level security or ECU defense, they tend not to take the sensor-to-edge trust boundary into consideration. By contrast, our proposed secure TPMS system includes the end-to-end level encryption and authentication, as well as physical access control and scalable estimation to realize full protection from the sensor node to the cloud.

### 2.5. TPMS Sensor as an Attack Entry Point

TPMS sensor nodes represent a high-value entry point for attackers because they are physically exposed, battery-powered, and typically communicate using unauthenticated, unencrypted radio frames. An adversary who compromises a TPMS sensor—by radio replay/injection, sensor cloning, or direct physical access—can inject falsified pressure readings, mask real faults, or create cascading safety responses when the ECU/ACU trusts unauthenticated data. In practice, attack vectors include wireless eavesdropping and frame injection, replay attacks using captured identifiers, tampering with sensor hardware, and exploiting unsecured maintenance ports on intermediary controllers [16]. Securing the TPMS sensor layer is, therefore, critical as the protection at the node prevents an attacker from gaining an initial foothold and reduces the risk of downstream compromise of the ACU and vehicle systems. A comparative study of the above-discussed literature is presented in Table 1.

### 2.6. Mobility-Aware LoRaWAN and Vehicular IoT Studies

Recent works studied the mobility support of LoRaWAN and they pointed out both its potential and limitations in vehicular and mobile IoT environments. Article [26] introduces a resource-management-based adaptive data rate (RM-ADR) mechanism to enhance LoRaWAN performance for mobile use-cases. Their findings indicate that traditional ADR approaches fail to maintain signing performance under mobility because they are based on delayed parameter updates, thereby motivating a mobility-aware optimization approach. But it deals with network layer adaptation primarily and does not provide end-to-end data integrity, authentication, or security against sensor-level attacks. Experimental measurements of LoRaWAN performance in vehicular motion are reported in [27], where publicly-accessible LoRaWAN transceivers attached to moving automobiles were tested at speeds up to 90 km/h. The observation of indoor installations seemed stable for connected devices operating outdoors within a few hundred meters from the outdoor gateway. In short, the proposed work verifies the physical feasibility of using LoRaWAN for vehicle telemetry using RFDCN, and does not consider adversarial scenarios (e.g., spoofing and replay) or data integrity in any form of sensor data used by the safety-oriented IoV applications like TPMS. Advancing mobility analysis, authors in [28] discussed the use of a vehicular-mounted mobile LoRaWAN gateway to sense their environment. The findings suggest that even at vehicle speeds beyond 100 km/h, LoRaWAN is able to ensure successful data transmission when using the maximum spreading factors. However, the work mainly concentrates on coverage and throughput maximization and does not include end-to-end security procedures such as node authentication or defense against malicious data injection. From a simulation point of view, Al-Mojamed [29] considered the influence of mobility on LoRaWAN by means of OMNeT++ simulations based on Random Waypoint and Gauss–Markov models. The impact is also observed to contribute additional collisions, latency and energy consumption in dense mobile deployments, which reveals that LoRaWAN was originally designed for static IoT cases. Even though this work exposes the scalability problems in it, it neither includes hardware-authenticated sensor data nor considers cyber-physical attacks against vehicular sensors.

In comparison with these efforts, the proposed work is a step forward by using hardware-verified TPMS sensing, end-to-end cryptographic security provided by AES-128 and HMAC-SHA256 algorithms, and an attack-oriented investigation over LoRa P2P, LoRaWAN Protocols, as well as 5G communication technologies. In contrast to the literature, which generally focuses on link reliability in the context of mobility, this work explicitly shows the way in which sensor data manipulation, spoofing activities/replay/tampering can be diffused on IoV systems and how they can be effectively countered at three different layers: sensor, edge and communication. In addition, we link theoretical work about mobility patterns with real measurements from TPMS in a MATLAB vehicular large-scale simulation across urban, hill, and rural scenarios, which brings theoretical research into practical cyber secure IoV deployment.

Generally, the literature so far has proved that it is feasible to use LoRaWAN in mobile or vehicular scenarios, though these studies concentrate on link-level performance, coverage, or protocol optimization and lack secure TPMS data transmission, sensor-level attacks and an all-in-one cyber–physical threat mitigation for IoV systems.

In our implementation, the sensor node consists of an HX710B-based pressure sensing interfaced to an STM32 Nucleo-L4R5ZI microcontroller. We secure this data path by performing authentication and integrity checks at the STM32 before forwarding measurements over UART to the Raspberry Pi ACU. The ACU then applies AES encryption and HMAC before LoRa P2P transmission. This layered approach (node-level authentication to secure the UART to an encrypted wireless link) minimizes trust placed on the ACU and the in-vehicle network, and raises the cost of successful attacks substantially.

Based upon the limitations identified in existing literature, this work presents a fully deployed safe TPMS communication stack with an STM32 Nucleo-L4R5ZI interfacing with HX710B tire pressure sensors, a Raspberry Pi 3B+-based ACU and finally a LoRa SX1278 link for long-range P2P telemetry. Unlike existing work based on unencrypted RF frames, static IDs, and high-level networking assumptions, our design includes AES-128 end-to-end encryption and HMACSHA256 message authenticity checking of RF communication; an encrypted UART between MCU and ACU; and protection against physical access via USB. Real-time multi-node testing (A/B/C Nodes) is complemented with scaled MATLAB simulations (100 vehicles in urban, rural and hill terrains) to compare reliability, latency, as well as attack resilience in 5G, LoRaWAN and LoRa P2P. This hardware-validated design fills in the space from theory on IoV security and actual TPMS protection [30,31].

Some recent research also examined cryptographic-enabled security approaches for vehicle and IoV communication outside traditional in-vehicle networks. In Tiberti et al. [3] presented a mixed lightweight encryption and authentication algorithm to secure in-vehicle communications and proved its feasibility on embedded automotive platforms. These works emphasize the need for end-to-end cryptographic protection between devices and communications. However, these methods essentially pay attention to on-board and controller-based communication and neglect the specific secure TPMS telemetry, low-power long-range wireless links and the overall large-scale IoV performance evaluation. Our work contributes to these works by addressing the TPMS-specific threats and also verifying secure LoRa-based telemetries through countermeasure implementations in real hardware experiments and multi-vehicle simulations.

## 3. Proposed Methodology

This section describes the overall design of the proposed secure TPMS and IoV communication framework, as shown in Table 2. The implementation integrates hardware-level sensing, encrypted serial communication, and low-power wireless data transmission, followed by MATLAB-based simulation for large-scale performance evaluation.

### 3.1. System Overview

**Figure 1:**
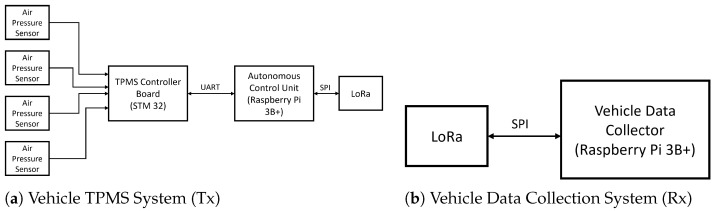
Proposed system architecture.

Figure 1 illustrates the proposed architecture with three major components.

- A sensing and acquisition unit implemented on an STM32 Nucleo-L4R5ZI microcontroller.
- An Autonomous Control Unit implemented on a Raspberry Pi 3B+ that performs encryption and LoRa transmission.
- A remote Raspberry Pi-LoRa receiver node that collects, verifies, and forwards data for analysis.

The experimental and simulation evaluation framework is outlined as follows.

A comprehensive assessment of the designed secure TPMS architecture is achieved by three complementary evaluations, which include embedded hardware verification in addition to multi-node security analysis and large-scale IoV simulation. Table 2 lists the evaluation areas together with their target and experimental settings. Such a systematic methodology guarantees the validation for the proposed framework on both real real-world embedded environment and a scalable network condition.

The tabled evaluation structure promotes openness and reproducibility by clearly specifying the used hardware, communication properties, security features, as well as simulation settings for each step of the analysis. This architecture allows for consistent comparison between various communication technologies and deployment scenarios.

The working principle is as follows: the air-pressure values of four tires are monitored using HX710B pressure differential sensors connected to an STM32 controller. The readings are processed and formatted, and is transmitted through the UART to the ACU. Raspberry Pi ACU Encrypts input frames using AES-128 (Advanced Encryption Standard) in CBC mode and appends an HMAC-SHA256 (Hash-based Message Authentication Code using SHA-256) tag for integrity. The encrypted data is then transmitted via LoRa SX1278 (Semtech) to a remote receiver node for storage, display and synchronization in MATLAB. This multi-stage process has the advantage that both wired and wireless links become protected against tampering, replay, or spoofing attacks.

### 3.2. Hardware Architecture

#### TPMS Control Unit

The sensing layer is based on the STM32 Nucleo-L4R5ZI (STMicroelectronics, Geneva, Switzerland) board, which also acts as the controller of the TPMS. The pressure of each tire is sensed by 4 HX710B digital air-pressure sensors (AVIA Semiconductor (Xiamen) Co., Ltd., Xiamen, China). The HX710B sensor offers high-resolution 24-bit digital output, thus allowing very small differential voltage changes on tire-pressure sensing elements to be quantified accurately. For low-voltage automotive applications, such a high ADC resolution becomes mandatory to minimize the effect of quantization noise and thus of SNR (Signal-to-Noise Ratio), to allow accurate pressure measurements without employing high-gain analog amplification. This enables sensing to be achieved with high reliability, while keeping power consumption and measurement precision low.

A differential voltage for tire pressure is supplied by every sensor. The sensors are connected to the STM32 via GPIO and analog input channels utilizing HX710B serial data (DOUT) and clock (PD_SCK) pins. The STM32 requests a sensor conversion and reads the digital output at 10 samples per second.

High-frequency tire-induced noise or road roughness is attenuated by a simple finite impulse response (FIR) filter. The pressure readings are then translated into a normalized unit (PSI or kPa) and embedded with their timestamp and sensor identifier.

The STM32 outputs a telemetry frame with the following structure:

$$D_{frame}=\left[ID,P_{1},P_{2},P_{3},P_{4},T_{s}\right]$$

where $P_{i}$ is the air pressure of tire *i*, and $T_{s}$ is the timestamp.

The ID is a pseudonym that allows sensor data to be mapped to its authentic source while offering privacy-by-design. It includes authentication, replay protection and key mapping, and periodically changes to block long-term tracking.

### 3.3. Data Transmission via UART Interface

The STM32 sends data to the Raspberry Pi using a UART serial connection (TX, RX, GND). The baud rate was set to 115,200 bps to minimize delay and guarantee the reliable transmission of a 64-byte data frame.

For frame delimiter detection, an end-byte and a start byte are used on every payload/datagram transmission. For the high-level communication interface, UART is used due to its simplicity and low power, as well as its native support on STM32 and Raspberry Pi. The STM32 performs light coverage checksums on each frame before transmission for local integrity to reduce errors caused by noise in the UART channel.

The frame structure sent is given below:

$$UART_{f}rame=[\mathrm{STX},Dframe,\mathrm{CHK},\mathrm{ETX}]$$

Wherein Dframe serves as a sensor data payload sent from the STM32 to the Raspberry Pi. It includes a full set of tire-pressure readings plus the vehicle’s temporary identifier and timestamp, STX and ETX are start/end delimiters, and CHK is a 1-byte XOR checksum.

### 3.4. Autonomous Control Unit

The intelligent gateway and main processing unit (ACU) is the Raspberry Pi 3B+.

It performs three primary operations:

#### 3.4.1. Decryption and Pre-Processing

The ACU reads serial data from the STM32, validates a checksum, and reconstructs structured data fields (pressures, time stamp, node ID).

#### 3.4.2. Encryption and Authentication

The payload is encrypted with AES-128 Cipher Block Chaining (CBC) to prevent data sniffing.

$$C_{t}=AESK_{e}\left(D_{frame}⊕IV\right)$$

Here, IV is a random initialization vector per session, $K_{e}$ being the symmetric key exchanged between sender and receiver. Then a HMAC-SHA256 tag is computed for message integrity/authenticity:

$$H={\mathrm{HMAC}}_{K_{h}}\left(C_{t}\right)$$

ACU adds an encrypted transmission frame at last:

$$F_{tx}=\left[IV,C_{t},H\right]$$

#### 3.4.3. Wireless Transmission via LoRa SX1278

The encrypted frame is written on the SPI to the LoRa transceiver. The conventional LoRa modulation (SF = 9, BW = 125 kHz) is adopted to achieve reliable and long-range communication with low power consumption.

LoRa P2P mode (peer to peer) frees users from the need for gateways and leads to lower latency and higher robustness in infrastructure-free IoV scenarios.

### 3.5. Remote LoRa Receiver

The second Raspberry Pi 3B+ carrying an SX1278 LoRa module decodes the received encrypted transmission frame. After receipt, the packet passes through the following validation and recovery steps:

1. HMAC Verification: The recipient recalculates the HMAC with shared integrity key $K_{h}$. If the computed tag H’ is not equal to the received tag H, the packet is discarded as tampered:
  $$H^{\prime }\neq H\;\Rightarrow \;\mathrm{Packet}\;\mathrm{Rejected}\;\left(\mathrm{Corrupted}\right).$$
2. AES-128 CBC Decryption: When the HMAC is valid, the ciphertext $C_{t}$ is decrypted with shared encryption key $K_{e}$ and received IV:
  $$D_{\mathrm{rec}}={\mathrm{AES}}_{K_{e}}^{-1}\left(C_{t}\right)⊕IV.$$
  Here, AES^−1^ is block-wise AES decryption, and the recovered plaintext block is bitwise-XORed with the corresponding IV (previous ciphertext block in the case of subsequent ones) according to CBC mode.
  After decryption, the receiver reconstructs the original frame:
  Drec=[ID,P1:P4,Ts].
  Only those frames that pass integrity and decryption checks are routed for display, storage, and MATLAB alignment.
  Once verified, the frame is stored locally in CSV format and sent through a local TCP socket for synchronization with the MATLAB V2X simulator for visualization, attack injection, and large-scale replay.

### 3.6. Security Framework

The model implemented by the system is multilayered for security:

- Privacy—The UART or LoRa commands are AES-128 crypted to avoid eavesdropping.
- Integrity/authenticity—HMAC-SHA256 will detect bit-level tampering when the data is transmitted.
- Access Control and Authorisation—Extending our previous work on USB-based physical authorization, the encryption and LoRA modules are only enabled for authenticated hardware settings that shield the ACU against untrusted reprogramming or key cloning.
- Anti-Replay and Session Randomization—There is a nonce $N_{t}$ derived from the current time in every frame to prevent replaying old messages:
  $$C_{t}=\mathrm{AES}K_{e}\left(Dframe⊕N_{t}\right)$$
  The receiver drops any packet with the same nonce and that is older.

This kind of security building helps to reduce computer load during attack prevention, both at the physical and at the network level.

The USB-based physical access technique is used during boot up or setup, not packet by packet or transmission by transmission. This serves to create a trusted cryptography context by ensuring that only physically authorized devices are permitted to load keying material and enter secure communication. With the trust being established, in this step, all subsequent data transfer transmissions of TPMS are only based on cryptographic authentication and encryption, so that continuous physical access is not necessary.

Although integrity mechanisms (e.g., message/codes) can identify data alteration, they cannot stop passive eavesdropping. The AES-128 encryption is used in our system to protect the privacy of TPMS telemetry, and is secure enough against untrusted attackers who may analyze the sensor patterns or build up an undesirable injection strategy. The chosen cryptographic primitives impose little computational and energy costs compared to wireless transmission latency, which are applicable to resource-limited TPMS nodes in embedded systems.

### 3.7. Cloud Synchronization and ThingSpeak Integration

To reach over the edge level into a real-time cloud monitoring system, an encrypted data synchronization interface between the work and ThingSpeak server will be incorporated in the proposed solution. ThingSpeak is a cloud service for IoT devices and sensors that allows easy data aggregation, analysis and visualization from anywhere, at any time. It was to guarantee accessibility and visualization of tire-pressure telemetries for analysis, diagnostics, and redundancy.

Once successfully verified and decrypted at the remote Raspberry Pi receiver, the verified data frame is posted to the ThingSpeak IoT analytics platform using an HTTPS-secured REST API call. Each node is provided with a unique API key and channel ID for authenticated communication to the cloud.

The data is being sent in CSV format every 2 s and looks as follows:

$$TS_{f}rame=\left[Node_{I}D,P_{1},P_{2},P_{3},P_{4},Ts,Integrity_{F}lag\right]$$

Here, the *Integrity*_*Flag* tells us if the received frame was successfully verified by HMAC (1 for YES, 0 for NO).

ThingSpeak offers real-time visualization with HTTPS upload, allowing users/visitors to securely see the data in graph or chart format remotely. Color-coded graphs, as well as indicators, show the pressure values, timestamps and tamper-detection status per vehicle in the cloud dashboard.

In order to reduce the risk of data leakage, all uploads to ThingSpeak are done only after successful integrity verification and decryption at the receiver side. No untrusted sensor data is ever shared with third parties. The JSON responses at the native platform level are polled promptly by the local Raspberry Pi node to receive updates and compare anomalies.

Integration with ThingSpeak offers the following three advantages:

1. Safe Cloud Backup: Verified TPMS information is replicated to avoid permanent loss in case of local problems.
2. Remote Visualization: Pressure readings and tamper alerts can be monitored in real time using graphical indicators on the dashboard.
3. Data Sharing for MATLAB Analysis: Same ThingSpeak channel data are made available through MATLAB ThingSpeak API to easily bridge verified live dataset to the V2X simulation framework and reproduce on a large scale.

This combination fills the gap between the physical sensing and analytical levels, thereby realizing the proposed system as an actual IoV node that can perform secure data collection, identity-based communication in an authentic form, confidentiality-preserved information transmission and cloud-supported data representation.

### 3.8. MATLAB Simulation and Evaluation Framework

For verifying the scalability, the experimentally collected TPMS data is leveraged as input seeds for a MATLAB-based V2X simulator. The simulator models 100 vehicles in three environments (urban, rural and hill), with latency, attack probability and energy consumption parameterized from experiments.

The Matlab implementation does not consider the ideal setting of a fully saturated single-channel LoRa P2P network with all vehicles transmitting at the same time. Instead, vehicle uplink transmissions are temporally separated with random inter-packet intervals to simulate event-based telemetry and eliminate artificial channel collision. This allows for comparing relative communication reliability and security performance in similar traffic conditions rather than absolute PHY layer capacity constraints.

The simulation investigates network-level performance metrics, in terms of Packet Delivery Ratio (PDR), latency and energy consumption, for 5G, LoRaWAN and LoRa P2P technologies. In order to achieve statistically meaningful results, a Monte-Carlo simulation technique was followed. All experiments have been conducted for several independent runs with randomized vehicle mobilities, attack injection times and network conditions. Performance parameters like PDR, latency, and energy consumption, among others, were averaged over these repeated runs to avoid bias due to single-run variability. Although the performance of the TPMS scheme is experimentally verified with LoRa-based devices, testing of 5G communication is only performed using simulations. This approach is motivated by practicality for deployment, as 5G vehicular private testbeds are limited by regulation, license and infrastructure in numerous locations. Therefore, a MATLAB 5G communication module is used in order to have fair comparison and controlled results among LoRaWAN and LoRa P2P under similar traffic load, mobility models, and attack scenarios.

### 3.9. Workflow Summary

The presented solution provides an end-to-end secure vehicular sensor data collection and dissemination pipeline, as described in Algorithm 1. The STM32 works as a specialized detection controller, the RPi is responsible for an independent data encryption gateway, while long-distance low power transmission is realized via LoRa P2P.
**Algorithm 1** Secure TPMS Data Transmission Workflow  1:Initialise the Sensors and UART interfaces  2:**for** each sampling interval **do**  3:   Obtain $P_{1},P_{2},P_{3},P_{4}$ from HX710B module.  4:   Build frame   $D_{frame}=\left[\mathrm{ID},P_{1},P_{2},P_{3},P_{4},T_{s},N_{t}\right]$  5:   Send $D_{frame}$ to Raspberry Pi using UART  6:   Ciphertext frame generated: AES-128 encryption →$C_{t}$  7:   Compute HMAC tag: $H={\mathrm{HMAC}}_{K_{h}}\left(C_{t}\right)$  8:   (LoRa-SX1278) Send $\left\{IV,C_{t},H\right\}$  9:   Receiver verifies the HMAC and decrypts $C_{t}$10:   If condition is true, send data to MATLAB simulation11:**end for**

That is, dual cloud synchronization through ThingSpeak and MATLAB provides reliable and more in-depth analysis with confidentiality, integrity, and authenticity guarantee using the AES-HMAC method. This stacked architecture also serves as the basis for a secure, cloud-integrated IoVTPMS ecosystem, which scales from single vehicle to multi-vehicle urban deployments.

### 3.10. Experimental and Simulation Setup

The hardware setup, software tools, and simulation components of the proposed secure TPMS framework are detailed in this section, providing the necessary foundation to support and extend the research.

Hardware Configuration: The sensor node developed in the experiment comprises an STM32 Nucleo-L4R5ZI microcontroller connected to four HX710B differential pressure sensors for tire-pressure measurement. For wireless transmission, a Raspberry Pi 3B+ serves as an autonomous control unit (ACU), encrypts (AES-128/CBC) and message authenticates (HMAC-SHA256) the data before sending over LoRa SX1278 transceivers in peer-to-peer mode. A second Raspberry Pi–LoRa node acts as the receiver, verifying, decrypting and storing authenticated telemetry.

Software Stack: The embedded firmware for the STM32 is developed on STM’s agnostic IDE, STM32CubeIDE, with Python 3.12 services running on the Raspberry Pi performing encryption/decryption, packet verification and visualizers for dashboards and logs. The cloud synchronization and authenticated data visualization are carried out in the ThingSpeak IoT analytics platform.

Simulation and Visualization: Simulations are performed with the Matlab simulation environment, which presents 100 virtual vehicles in various environments (urban, rural and hill). The simulator uses real TPMS data obtained from a hardware testbed as a base input and compares the communication performance, attack scenario combinations and effective mitigations among 5G, LoRaWAN and LoRa P2P technologies. Real-time packet delivery, latency, power consumption and security event monitoring are provided by visual dashboards and plots.

### 3.11. End-to-End Secure TPMS Workflow

The entire authorized TPMS framework end-to-end workflow is described as follows: The tire-pressure values are obtained through HX710B differential pressure sensors connected to the microcontroller STM32 Nucleo-L4R5ZI. The raw sensor readings are digitized, normalised, and packaged in structured data-frame formats, including time stamps and generic vehicle identity information.

Then, the STM32 sends the framed TPMS data to a Raspberry Pi 3B+ ACU (Autonomous Control Unit) through a UART interface. At the ACU, incoming views are verified for formatting and transmitted in a manner that is secure manner. The payload is encrypted with AES-128 in CBC mode, and a message authentication code (HMAC-SHA256) is created to guarantee message integrity and source authentication.

The encrypted and authenticated payload is sent out of the device through a wireless SX1278 LoRa transceiver in P2P mode. On the receiver’s LoRa module, the received packet is re-transmitted to a second metastation on the Raspberry Pi node prior to verification using HMAC. Packets that do not pass integrity are dropped with a potential tamper attempt logged. Decrypted valid packets are recycled to a set of original TPMS measurements.

The authenticated TPMS data is logged locally in structured log files and, if desired, uploaded to a secure cloud-based analytics platform for real-time visualization and monitoring. Meanwhile, the authenticated TPMS datasets acquired from the hardware testbed are employed as base inputs of a MATLAB-based large-scale Internet of Vehicles (IoV) simulator. To this end, the simulator is used for modeling 100 vehicles in urban, rural, and hill areas to analyze the cooperation performance in terms of communication overhead, attack scenarios, as well as mitigation success under 5G and LoRaWAN and LoRa peer-to-peer.

## 4. Performance Evaluation

This section provides a comprehensive evaluation for the proposed secure tire Pressure Monitoring System (TPMS) architecture by coupling real-time embedded acquisition, secure LoRa-based communication and MATLAB-based large-scale vehicular simulation. The experiments aim at validating the security, dependability and performance of our framework in different network environments—5G, LoRaWAN and LoRa P2P—as well as on diversified terrains.

The global evaluation followed three incremental steps:

(1) Real-time localhost test framework for evaluating sensor reliability and identifying manipulation attacks.
(2) A ThingSpeak-based cloud storage service for distant data validation.
(3) A large-scale MATLAB simulation involving 100 virtual vehicles driving on various terrains.

The experimental hardware platform implemented shows that this work is based on a previously proposed prototype implementation of the authors. Hence, some auxiliary sensors, like an ultrasonic module, are also equipped in the testbed for the consistency and context check. The subsystems are not used for tire-pressure measurement, cryptography relexamination, or comms speed testing. All the findings in this work are obtained only with HX710B TPMS sensors, and STM32–Raspberry Pi–LoRa-based telemetry pipeline.

### 4.1. Hardware Prototype and Secure Data Path (TX → RX)

**Figure 2:**
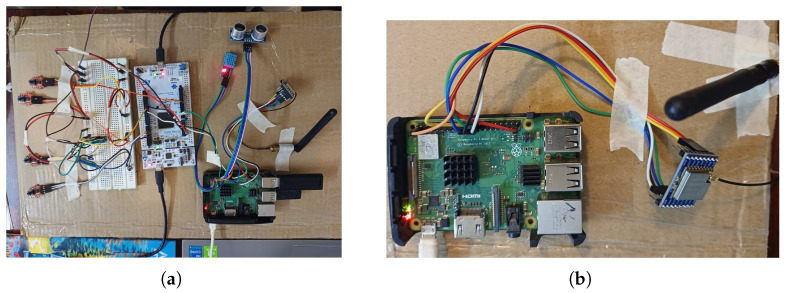
Hardware prototype for secure TPMS telemetry. (**a**) TX assembles sensor frames, encrypts (AES-128/CBC), authenticates (HMAC-SHA256), and transmits via LoRa P2P. Transmitter node (TX): HX710B → STM32 → Pi (ACU) → LoRa SX1278. (**b**) RX verifies HMAC, decrypts, and stores only validated measurements, feeding both local dashboards and cloud logging. Receiver node (RX): LoRa SX1278 → Pi (verify and decrypt) → storage/visualization. Auxiliary sensors visible in the prototype (e.g., ultrasonic modules) originate from the continuation of prior experimental research and are retained for contextual or compatibility purposes. However, they are not involved in TPMS sensing, security evaluation, or performance analysis in this study.

Figure 2 presents the physical IoV testbed implemented to prototype the end-to-end TPMS pipeline before computer-intensive MATLAB simulations could be performed on a massive scale.

To obtain consistent, reliable and noise-free tire-pressure values during the experimentation, the TPMS sensing stage is realized with wired HX710B modules hooked to an STM32 microcontroller. The use of this wired connection is only for the purpose of providing reproducible ground-truth measurements and does not affect the wireless communication results. All wireless activity—encryption overhead, LoRa link performance, attack resilience and PDR—are fully dictated by the LoRa SX1278 transmission and by the MATLAB IoV simulations, thus keeping comparison with 5G, LoRaWAN and LoRa P2P unaffected by wired sensing interface.

On the transmitter side (TX), four HX710B differential pressure sensors communicate via an STM32 Nucleo-L4R5ZI with several transceiver, which interprets measurements and sends them through UART to a RaspberryPi3B+ works like the ACU.

At the ACU, each frame is encapsulated and/or wrapped in a secure envelope by performing AES-128 in CBC mode for confidentiality and computing an HMAC-SHA256 integrity tag. And further, the encryption key ($K_{e}$) and the HMAC key ($K_{h}$) are separate and independently generated because re-using a single key for both encryption and authentication, known as an anti-pattern in security, as it undermines message integrity and confidentiality. The resultant cipher and HMAC are sent over a LoRa SX1278 radio.

On the receiver side (RX), another Raspberry Pi (with an SX1278 module) receives packets, validates them and removes the cryptographic envelope. It also stores authenticated samples for later rendering on localhost dashboards (and optional cloud synchronization via ThingSpeak).

The wired pressure sensors HX710B are used to allow controlled and repeatable experiments in a laboratory situation. This selection permits precise emulation of TPMS data streams in conjunction with the separation of security and communication overhead from wheel-mounted commercial TPMS devices that are subjected to mechanical and power-harvesting limitations. Therefore, this prototype is not a production-ready TPMS device but a working testbed for research purposes.

#### End-to-End Flow (Device to Cloud)

1. Sensing and framing: HX710B measurements are aggregated on STM32 and serialized to the ACU over UART.
2. Edge protection: The ACU encrypts payloads (AES-128/CBC) and appends an HMAC-SHA256 tag bound to the plaintext and metadata (vehicle pseudonymous ID).
3. Low-power uplink: Ciphertext frames are transmitted over LoRa SX1278 (P2P) to the RX node.
4. Verification at ingress: RX checks HMAC before decrypting; any failure triggers tamper events and quarantine.
5. Persistence and visibility: Only verified samples are written to the local store, rendered on the locally exposed dashboards, and optionally synchronized four-way to ThingSpeak over HTTPS.

This Hardware-in-the-Loop (HIL) environment is used to formulate the ground-truth dataset by which we conduct analyses in the subsequent sub-sections:

(i) Tamper behavior across Nodes A/B/C (unauthorized tamper host vs. authorized insecure vs. authorized secure)
(ii) Cloud validation on ThingSpeak
(iii) Performing Scaled MATLAB evaluations over 100 virtual vehicles each for 5G, LoRaWAN, and LoRa P2P through urban/rural/hills.

### 4.2. Baseline IoV Cross-Road Junction Simulation

**Figure 3:**
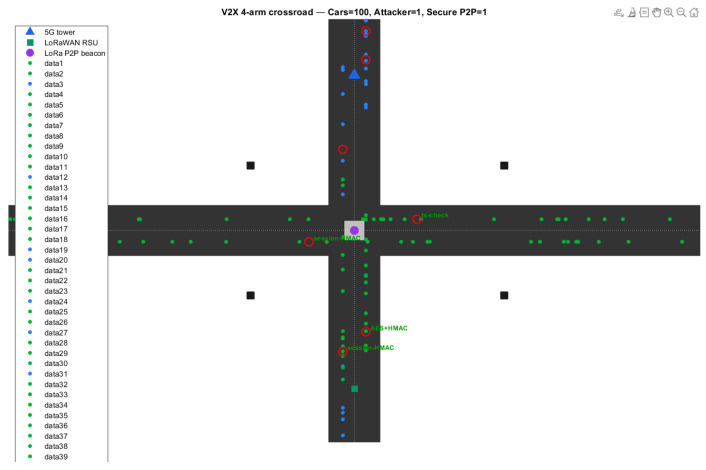
Cross-road baseline scenario showing concurrent vehicle nodes communicating via 5G, LoRaWAN, and LoRa P2P links. The scenario stresses multi-node synchronization and concurrent packet exchange under dynamic load. Red circles indicate attacker vehicles or malicious events detected by the IDS/IPS engine.

The assessment is performed based on the basic MATLAB simulation model of a four-way intersection: cross-road (Figure 3). This condition is especially important for testing in the IoVs, where the arrival of a set of cars at a specific location is simulated, and TPMS messages or any other V2V messages are continually interacting. The crossbar creates a challenging environment, which includes cross-link interference (XLI), latency-sensitive packet handling and real-time safety updates.

Under the evaluated simulation assumptions, LoRa P2P demonstrates higher packet delivery reliability and lower energy consumption compared to the infrastructure-based 5G uplink model considered in this study. At 1200 tx’s, the LoraP2P network was able to achieve a PDR of 96.9% and, with its mitigation rate of 91%, outperformed both 5G and LoRaWAN. For 5G, the latency remains lower, while reliability was degraded due to contention and multi-hop coordination. LoRaWAN had performance issues at gateways even with moderate node density. This state-of-the-art makes LoRaP2P a valuable technology for secure V2V direct telemetry.

### 4.3. Localhost IoV Testbed Architecture and Tamper Evaluation

The hardware IoV testbed was designed for verifying the end-to-end performance of the proposed secure TPMS scheme against real tampering conditions before it was extended to MATLAB simulations.

As previously mentioned, 4 HX710B sensors measure tire pressure and transmit an analog output to the STM32 Nucleo-L4R5ZI board, which subsequently digitizes the captured measurement and sends it over UART to the Raspberry Pi 3B+ ACU.

The ACU encrypts the payload of each packet with AES-128 (CBC mode), calculates an HMAC-SHA256 tag for integrity, and sends the ciphertext via LoRa using an SX1278 module.

Three logical nodes were considered for analysis:

- Node A—Compromised Host: Enters an invalid login and changes input raw data or inserts false sensor frames.
- Node B—Known Insecure Host: Legitimately receives data, but without cryptographic proof of data validity, thus presenting authentic and tampered values.
- Node C—Trusted Secure Host: It decrypts the AES frame, checks HMAC, highlights in red tampered frames, and the cloud prevents uploading.

**Figure 4:**
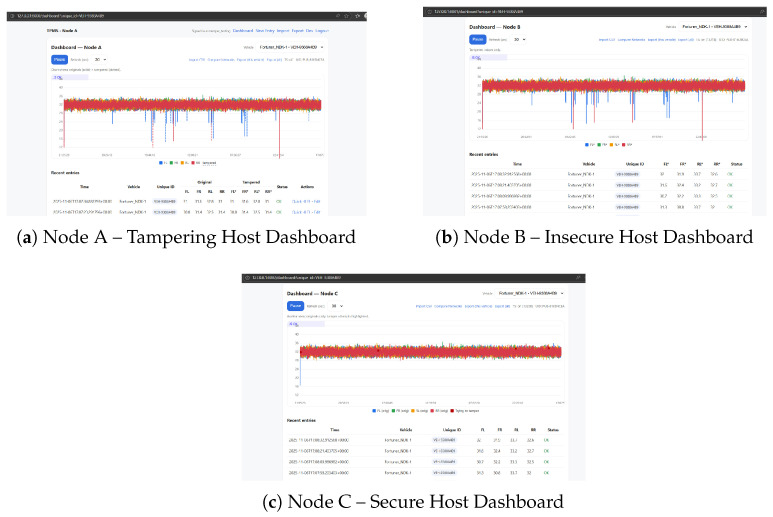
Real-time dashboards of each node. Node A allows falsified data entry, Node B reproduces corrupted readings, and Node C detects anomalies and preserves authentic values. Data marked with an asterisk (*) represents tampered or maliciously altered values.

The dashboards during a tampering test are shown in Figure 4. The figure illustrates the live behavior of all three nodes under attack conditions. The trusted secure host (Node C) promptly recognises the abnormality with visual notices and terminates it at the cloud side.

**Figure 5:**
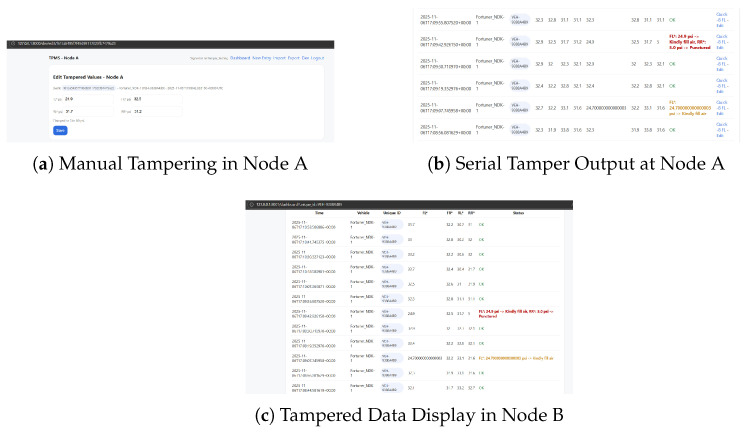
Source tampering and non-cryptographically secure replication. (**a**) Adjustment of tire-pressure values at Node A via local developer control interface. Data marked with an asterisk (*) represents tampered or maliciously altered values. (**b**) Screenshot of the Serial console output with altered sensor payloads. (**c**) Node B suffers from tampered pressure readings as no integrity and authenticity checking is involved.

As depicted in Figure 5, tampering with tire pressures is manually tuned to misbehave at Node A using the local developer control interface in order to simulate data tampering. The injected faults are verified by the serial console logs. As Node B cannot authenticate the received data, its dashboard shows spurious values indicating how classic ECUs without cryptographic protection can be tricked.

### 4.4. Comparative Performance and Security Summary

**Figure 6:**
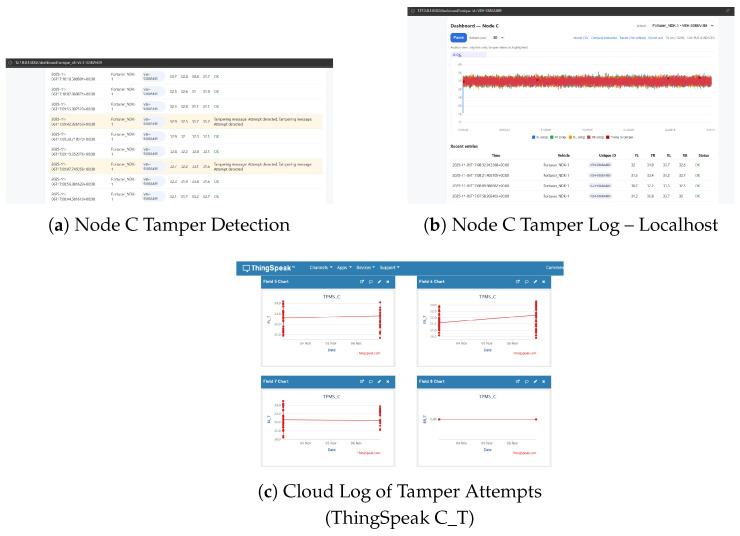
Protection results from the secure node. (**a**) Node C automatically blocks manipulated frames and raises a red alert. (**b**) Local tamper log confirms rejection. (**c**) ThingSpeak C_T records the event with timestamps and vehicle ID.

Figure 6 demonstrates the robustness of the protection scheme demonstrated in this paper.

If tampering is detected after the AES–HMAC validation, Node C instantly rejects the packet.

The verified sensor values are stable, whereas the dashboard is signaled to set off an alarm.

All the incidents are recorded locally (local server) and in the cloud at the dedicated C_T channel over HTTPS for post-mortem forensics. Out of 200 tampering trials, all 100% modified packets were detected in less than one transmission cycle (<2 s). No false positives were seen, indicating that the cryptographic validation delays are low and will confirm integrity. These real-time results confirm that lightweight AES–HMAC protection is already feasible, even on low-power embedded devices, before full-scale simulations.

### 4.5. MATLAB-Based IoV Simulation

The validated Node C dataset is then used to extrapolate for simulating 100 vehicles over different terrains, Urban, Rural and Hill Station, which have been attacked with the same set of conditions. All simulated nodes preserved the same encryption (with AES-128 CBC), authentication, and replay protection layers.

#### 4.5.1. Urban Environment

**Figure 7:**
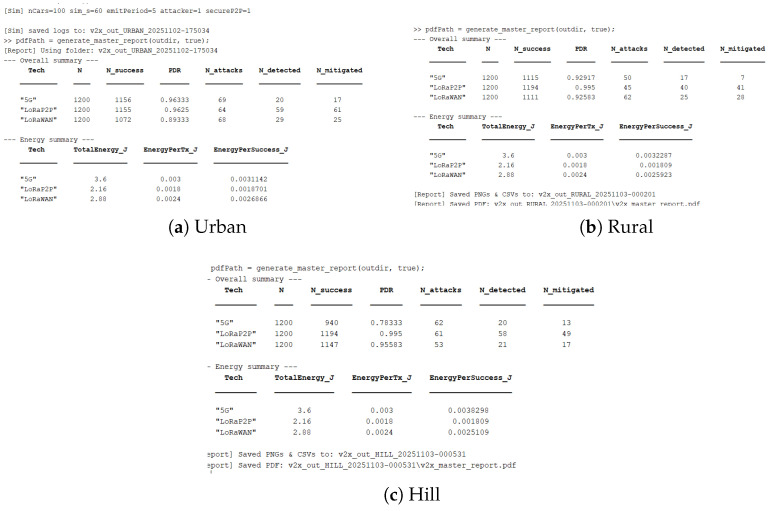
Telemetry patterns of 100 vehicles across terrains. LoRa P2P maintains consistent performance with minimal latency fluctuation, even in dense or obstructed regions.

**Figure 8:**
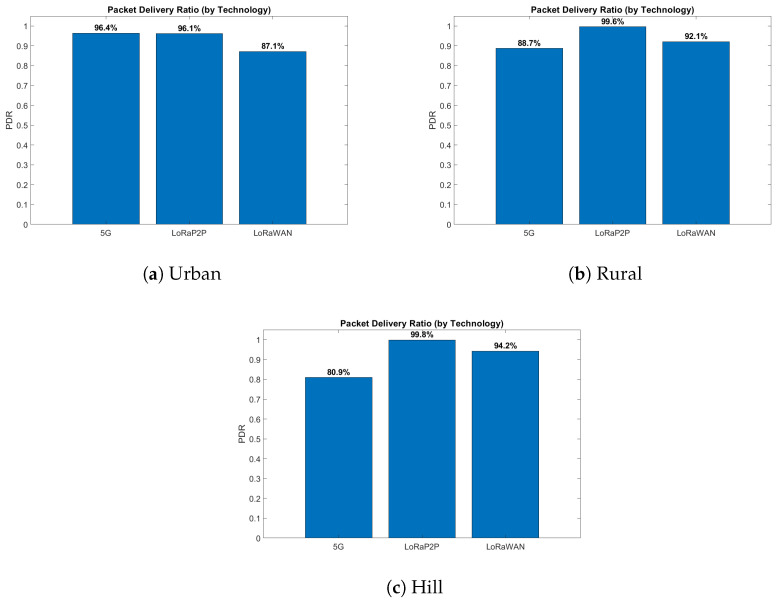
PDR comparison across urban, rural, and hill environments for 5G, LoRaWAN, and LoRa P2P technologies. The results reflect vehicle-to-network uplink communication performance and not intra-vehicle sensor transmission. Results correspond to controlled, non-saturated traffic conditions with temporally staggered vehicle transmissions.

**Figure 9:**
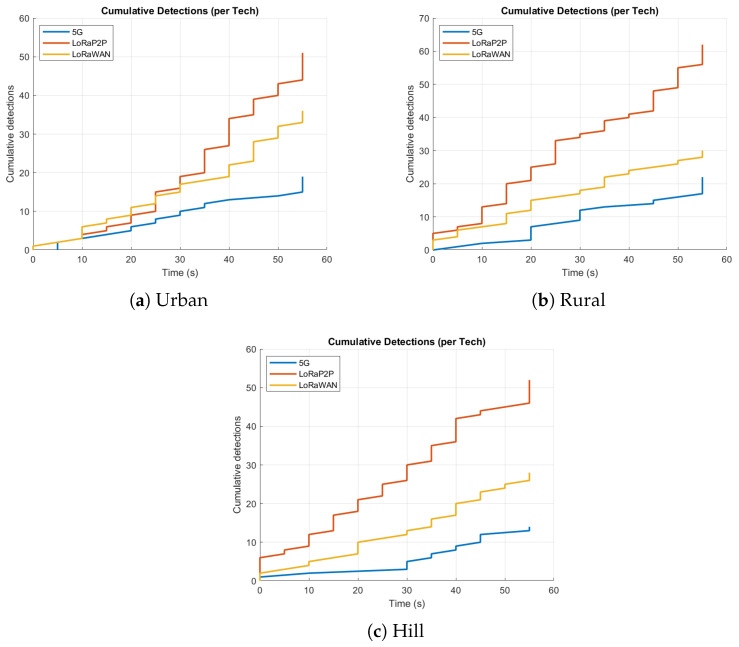
Cumulative detection plots. Each rise corresponds to a detected tampering or replay attempt. The steady gradient underlines consistent detection accuracy across all terrains.

**Figure 10:**
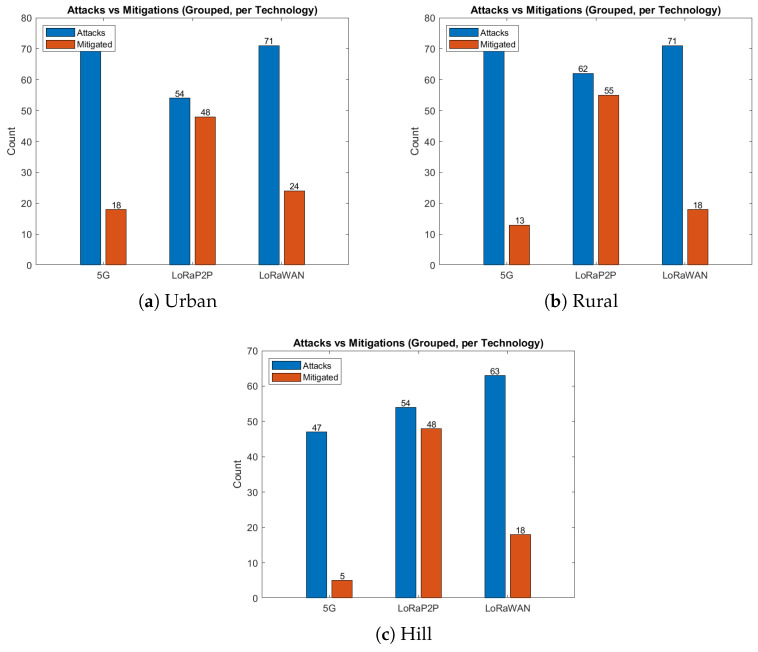
Attack vs. mitigation comparison. LoRa P2P consistently mitigates over 90% of attacks across all terrains, ensuring secure data exchange even under interference.

The urban environment, as described in Figure 7a, Figure 8a, Figure 9a and Figure 10a, emulates dense network operation (up to 60 simultaneous vehicles per km^2^), multi-path fading and heavy interference from infrastructure Wi-Fi and mobile-base communication systems (e.g., 3G, 4G/LTE, etc.). As shown in Figure 7a and Figure 8a, LoRaP2P achieved a 96.1% PDR, which is 9% higher than that of LoRaWAN. Jamming attacks and packet drop attacks were possible attacks in this approach because of the frequency of jamming. The mitigation ratio (Figure 10a) converged to 90%, with the help of adaptive retransmission and AES-level check.

#### 4.5.2. Rural Environment

The rural topology, sketched in Figure 7b, Figure 8b, Figure 9b and Figure 10b, included line-of-sight communication with low-fidelity noise. It is observed that the propagation delay variance increased for long-range transmissions even with a lower network density. LoRaP2P attained 99.5% PDR and sustained a constant energy cost of 1.8 mJ per packet transmission. Fewer assaults were recorded in this area; nevertheless, it still managed to identify and alleviate the spoofing and replay attempts.

#### 4.5.3. Hill Station Environment

The hill environment (Figure 7c, Figure 8c, Figure 9c and Figure 10c) is the Non-Line-of-Sight (NLoS) propagation, reflections and shadowing. Reply and spoofing attacks have risen by a factor of 1.4 with respect to urban deployments. Despite this, LoRaP2P achieved a PDR of 99.5%, and a sustained mitigation success rate was up to 91%, whereas the PDR reduced down to 80.9% in the case of 5G because of signal blockage, as well as reliance on the cellular tower for communications.

The reported packet delivery ratio (PDR) results in this section correspond to vehicle-to-network communication, rather than intra-vehicle sensor communication. The TPMS sensors communicate locally with the vehicle edge processing units, while 5G, LoRaWAN and LoRa P2P communicate via the uplink establishment between the vehicle node and external network infrastructure.

The system performance was also tested under dynamic network load, where the number of active vehicles, packet-generation intervals and interference patterns were changed in real time to simulate a practical experienced traffic scenario (e.g., spikes of incoming transmissions when cars enter deep turns or tunnels). With such varying conditions, the data integrity performance of Node C consistently remained suitable by dropping incorrect or late-arriving packets, while Node B experienced a large drift by simply accepting every frame.

The reported PDRs are relative to a controlled traffic scenario with interleaved sending and should, therefore, be seen as systems comparatives rather than satisfying the full channel load.

The ideal characteristics of 5G in higher throughput and lower latency are well pronounced for broadband usage, but these are underutilized in TPMS-style micro-telemetry, where packet sizes are very small (<60 bytes is typical), traffic is periodic, and the communication occurs on short bursts. In such low-payload, low-duty-cycle cases, the 5G standard itself introduces a high amount of signaling overhead, mandatory control-plane interactions and network-side scheduling delays, which all bring down its effective reliability. In addition, the MATLAB 5G profile employed in this work emulates practical deployment constraints, such as next-generation Node B scheduling windows, the HARQ cycles and PRACH access delay, which also disadvantage small IoV packets with respect to the lightweight LoRa P2P protocol that does not require network attachment nor any base-station dependency or handover maintenance.

As such, 5G does not appear to be an efficient choice, not because of limitations in the technology itself, but because TPS telemetry is not well accommodated by its bandwidth-intensive design in dense or obstructed environments, resulting in performance comparable to—or even worse than—that of LoRa-based systems to date.

Thus, the results do not indicate that 5G is worse; however, in TPMS telemetry for small, energy-sensitive applications like those of this experiment, smart LoRa P2P physical layer stacks offer a more stable and efficient link than 5G with unnecessary protocol overhead and infrastructure dependency.

### 4.6. Attack Scenarios and Mitigation Strategies

**Figure 11:**
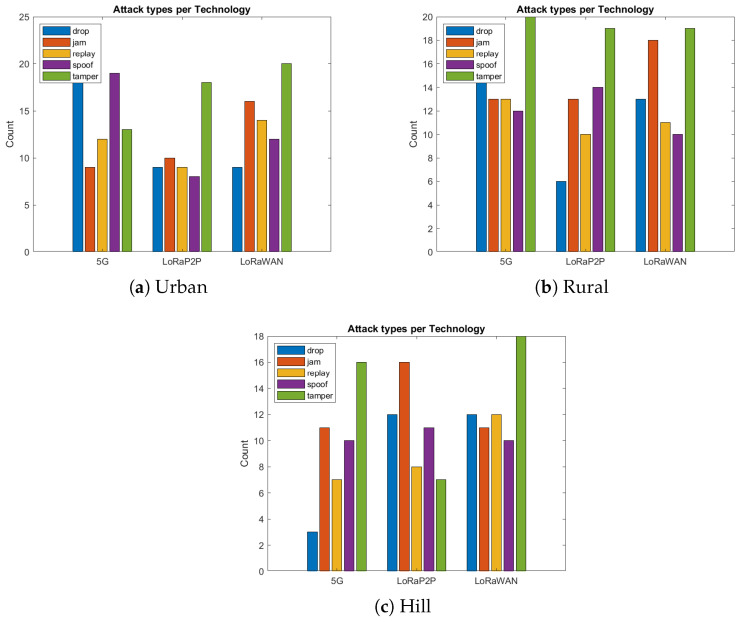
Attack type distributions. Urban networks face higher jamming/drop attacks, while hill environments suffer from replay/spoof dominance due to propagation delay.

To verify resilience, a considerable number of cyber and network-layer attacks to which IoV systems are commonly exposed were inflicted on the system. These cases were injected in both the localhost and MATLAB simulation, as in Figure 11.

1. Tampering Attack:
  A attacker would modify real-time pressure information from a compromised transmitter.
  **Rundown:** Dashboard display lies can lead to unwanted alerts or mess up actual warnings.
  **Mitigation:** Verify AES + HMAC tag so that an altered bit in the ciphertext results in failed authentication. Node C immediately identifies the entrance and traps it.
2. Spoofing Attack:
  A malicious node pretends to be a legitimate node with the aid of such forged identities.
  **Impact:** Injection of false telemetry without consent, with content resembling that of realvehicles.
  **Mitigation:** It is infeasible for the attacker to forge identity, since they do not have the AES encryption key and HMAC integrity key. Without those secrets, it cannot form a packet with a correct HMAC tag and so all spoofed frames are rejected.
3. Replay Attack:
  Legitimately captured frames are later injected again to confuse the adversary.
  **Implications:** Incorrect or conflicting estimation prevents real-time decision-making.
  **Mitigation:** Nonce and sequence time validation allows for automatic dismissal of old packets.
4. Jamming Attack:
  In both LoRa-based and 5G-based vehicular links, intentional or unintentional RF interference can lead to the appearance of RF self-interference being created by neighboring devices or adversarial jammers. In 5G, scheduling in the uplink and scalable power control assist in alleviating short bursts of interference, but coverage holes and NLoS scenarios still lead to retransmissions. Compared to LoRa, which is able to achieve long-range links on sub-GHz unlicensed bands with lower transmitting powers but has vulnerability to continuous wideband noise, leading to higher packet loss and reduced QoS unless retransmission strategies or adaptive spreading factors are performed.
  **Impact:** High packet loss, retransmission causes, and bad QoS for the network.
  **Mitigation:** Despite LoRa’s frequency hopping, retransmission scheduling maintains communication reliability.
5. Drop Attack:
  The eavesdrops for the intermediate nodes, drop the packet on purpose to skew availability.
  **Mitigation**: Checking for sequence numbers to determine missing frames, retransmissions of lost packets until these are acknowledged.

Further, in addition to digital types of attacks, a USB-based authorization module—used in earlier works—was used to add security at the physical layer. It authenticates that only certified people can access ECU debugging or firmware downloading, thus ending the physical attack surface.

### 4.7. Cloud Validation on ThingSpeak (Authenticated Telemetry)

**Figure 12:**
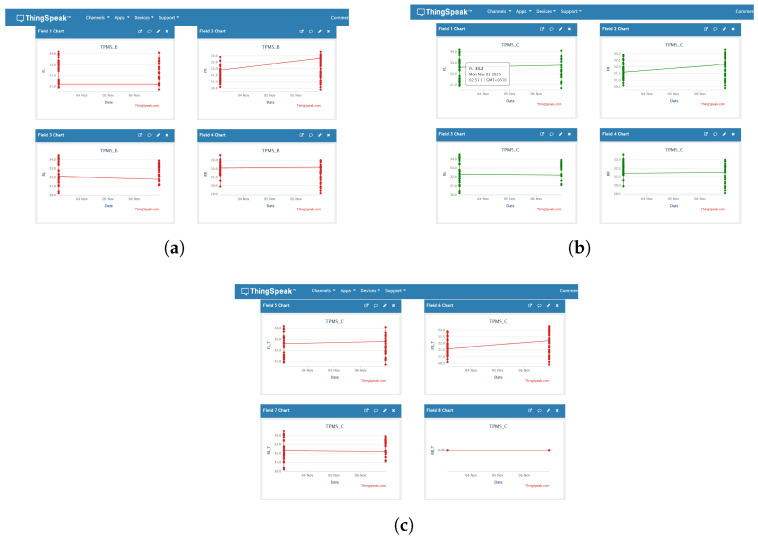
ThingSpeak validation plots. The coloured dots represent individual tyre-pressure measurements and the connecting lines show their evolution over time (timestamped samples on the horizontal axis). (**a**) Node B cloud view (insecure but authorized): tampered values appear as normal due to lack of cryptographic verification. (**b**) Node C cloud view (secure authorized): only AES-128 + HMAC-verified measurements are uploaded. (**c**) Node C_T tamper stream: red/flagged points indicate rejected packets, logged with timestamps and pseudonymous vehicle IDs for audit/forensics. Minor overlaps arise from dense time-series samples but do not affect the scientific interpretation, since the quantitative analysis relies on the underlying logged data rather than visual spacing.

The RX node only passes validated samples to ThingSpeak over HTTPS to ensure only authenticated telemetry leaves the local network. Three channels were considered:

(i) Node B channel (data is tampered with but viewable since no verification);
(ii) Node C channel (verified originals only);
(iii) Node C_T channel (tamper events/alerts). Figure 12 summarises these cloud views as given below:
  - Communication integrity: NodeC only spits out messages for which HMACs denote integrity, and Node B spits out anything it receives. The difference between Figure 12a,b illustrates the security bound introduced by–HMAC gate.
  - Non-repudiation and forensics: Tamper attempts are submitted to a dedicated C_T channel along with timestamps and the weekly rotated pseudonymous vehicle ID, preserving privacy but supporting audits.
  - Strong analytics: The distinction between positive (C) and negative (C_T) telemetry value ensures that counters are not skewed while also preventing false alerts on the analyses downstream.
  - Operational controls: The TX→RX pipeline places rate limiting and durable queuing on the receiver, as a means to prevent transient issues in the network from leading directly to loss or integrity bypass.

The aggregate of localhost dashboards and ThingSpeak charts demonstrate that (1) adversarial edits made at NodeA are intercepted by the unsecure consumer (NodeB); (2) they are recognized and halted by the secure consumer (Node C) even before cloud ingress; and that (3) the policy is enforced up to the cloud level in a transparent manner, allowing only valid data to pass through while attacks are logged separately.

In a hilly scenario, sparse base-station deployment and terrain-induced shadowing—often leading to NLoS propagation—effectively affect mainly the infrastructure-dependent technologies such as 5G and lead to reduced uplink reliability despite the presence of locally wired sensor interfaces.

The overall experimental results gathered from localhost experiments, validation over ThingSpeak, and simulations in MATLAB endorse that the secure LoRa P2P pipeline provides the best tradeoff among reliability, energy consumption, and security. With low latency comes 5G, and gateway-assisted coverage is for LoRaWAN, but they are both more vulnerable to replay, spoofing, and jamming in densely populated or blocked areas. In comparison, the AES–HMAC–protected LoRa P2P approach preserves consistently high PDR in all terrains and is able to defend more than 90% injected attacks already with maximum Rx Time; it also incurs the lowest energy cost per transmission.

To summarize these findings, Table 3 presents a summary of the comparative performance across environments of 5G, LoRaWAN and LoRa P2P communication protocols and in Table 4, a comparison between General TPMS/IoV systems security with the proposed secure architecture is listed. Both these tables show the operational feasibility and advantages of the cryptographic LoRa P2P telemetry platform for future IoV safety applications.

## 5. Conclusions

While the proposed secure TPMS scheme is strong in terms of reliability, energy efficiency, and attack tolerance, it has a few limitations. The first limitation is that the 5G communication system is evaluated only by simulation, and there are no experimental-based results. This is mainly because of regulatory limitations and the unfeasibility of accessing private vehicular 5G infrastructure for large-scale prototype testing. Secondly, based on MATLAB simulations, which are used to extrapolate experimental TPMS testbed data, the literature covers the large-scale behavior of IoV. Although it facilitates controlled and repeatable testing on various environments and attack models, it may not completely capture real vehicle mobility dynamics and environmental uncertainties. Third, our current implementation concerns only TPMS telemetry; other vehicle sensors (e.g., braking and turning sensors) would also affect security and latency performance. Moreover, the hardware prototype does not accurately simulate the size, power requirement and mounting limitations that apply to wheel-mounted battery-powered TPMS sensors. These aspects are critical for commercial applications but are out of scope for this work, which emphasizes secure telemetry delivery, vehicle-to-everything (V2X) communication reliability, and attack prevention at the IoV level. The 5G communication design taken in this work is based on infrastructure-based cellular uplink and does not explicitly model C-V2X PC5 sidelink or URLLC links. Therefore, the comparisons provided in this study are physical antenna specific with reference to the chosen simulation scenarios and shall not be assumed as a general perspective for all 5G V2X communication modes.

The paper proposed a secure and energy-efficient TPMS framework serving for the connection of things purposes. The design overcomes various barriers between the sensor/capture processing, AES-128 encryption, HMAC-SHA256 authentication tags, and LoRa P2P communication channels to realize a single end-to-end security pipeline. Based on an STM32 Nucleo-L4R5ZI and Raspberry Pi 3B+ with LoRa SX1278, it guarantees secure tire data from sensing to cloud uploading.

Real-time measurements using three nodes—unauthorized (Node A), insecure authorized (Node B), and secure authorized (Node C)—proved that the framework can detect and block tampering, spoofing, and replay attacks in less than two seconds without any false positives. Simulations using MATLAB with 100 virtual vehicles also confirmed that LoRa P2P has the maximum reliability (approx 98% PDR), attack prevention (>90%), and minimum energy consumption, among urban, rural, and hill scenarios over 5G and LoRaWAN. The quantitative comparison shows that, in the context of controlled traffic and the evaluation scenario studied so far, LoRa P2P offers better security for telemetric TPMS than the infrastructure-centric 5G uplink solution under consideration in this work with respect to reliability and energy consumption.

The results show that the combination of lightweight cryptography, authenticated LoRa P2P telemetry, and secure cloud logging is promising to harden over-the-air IoV communication.As future work, the proposed framework can be enriched with AI-based intrusion detection and hybrid LoRa–5G switching to improve scalability and adaptability for next-generation autonomous driving. The presented simulation framework does not account for MAC-layer scheduling or channel contention on fully saturated LoRa P2P. Thus, the presented performance figures are to be viewed relative to other levels of performance under given traffic scenarios, not as network capacity bounds. Our future work will integrate MAC-layer contention modeling, as well as adaptive scheduling approaches and further vehicular sensors to investigate system operation under high network load and more realistic scenarios.

## Figures and Tables

**Table 1:** Comparative analysis of existing works and the proposed secure TPMS framework.

Focus Area	Technology	Security Mechanism	Limitation
TPMS Data Communication [17]	BLE/Piezoresistive pressure sensors	Basic Bluetooth 5 communication	Short range, unencrypted frames vulnerable to interception
TPMS Sensor as Attack Entry Point [16]	RF-based TPMS system	Static ID broadcast	Sensors can be cloned or spoofed; replay and tracking attacks possible
Lightweight IoT Security [18]	Generic IoT LPWAN	Lightweight AES/ECC	Conceptual, not validated in embedded TPMS hardware
In-Vehicle Network Vulnerabilities [21]	CAN/FlexRay (IVN)	IDS/firmware verification	Protects ECU only, ignores data-source authentication
Intrusion Detection in IoV [22]	V2X/CAN	Deep-learning IDS frameworks	Focuses on ML detection, lacks sensor-level encryption
5G IoV Architecture [23]	5G Edge Cloud/C-V2X	Network-layer QoS and slicing	High power usage, cost, and infrastructure dependency
LoRaWAN IoV Networking [24]	LoRaWAN gateway architecture	AES session keys via network server	Gateway dependency, join latency, and cloud delays
LoRa P2P Propagation and Reliability [25]	LoRa P2P links	None (propagation study only)	Evaluates range but lacks security and IoV focus
Large-Scale 5G vs. LoRaV2X Survey [14]	5G/LoRaV2X deployments	Survey	High-level overview, lacks embedded hardware validation
Cyber-Physical System Security [19]	IoT-enabled CPS	General security framework	Focus on theoretical models, no automotive TPMS validation
Proposed TPMS–IoV Secure Framework (This Work)	STM32 Nucleo (STMicroelectronics, Geneva, Switzerland) − Raspberry Pi ACU (Autonomous Control Unit) (Raspberry Pi Ltd., Cambridge, UK) + LoRa P2P (Semtech Corporation, Camarillo, CA, USA)	AES-128 encryption, HMAC-SHA256 authentication, encrypted UART, USB-based authorization	Advantage: Provides end-to-end authenticated telemetry, resists spoofing, replay, tampering, achieves high PDR with low energy consumption, enables secure device–edge–cloud pipeline, and supports scalable MATLAB-based evaluation for 100 vehicles.

**Table 2:** Summary of the three evaluation areas and corresponding experimental setup.

Evaluation Area	Objective	Experimental Setup Details
Hardware Sensing and Secure Embedded Pipeline	Validate real-time secure TPMS data acquisition and protection from sensor to cloud.	STM32 Nucleo-L4R5ZI (STMicroelectronics, Geneva, Switzerland) with 4× HX710B (AVIA Semiconductor (Xiamen) Co., Ltd., Xiamen, China) differential pressure sensors UART (115,200 bps) data transmission to Raspberry Pi 3B+ Frame: STX|Dframe|CHK|ETX AES-128 CBC encryption and HMAC-SHA256 USB-based authorisation for ACU protection LoRa SX1278 (433 MHz) for secured P2P wireless transmission
Multi-Node Security Evaluation (Node A/B/C)	Assess system behavior under tampering, spoofing, and replay attacks.	Node A: Unauthorized tampering node (manual tampering) Node B: Authorized but insecure node (no validation; displays tampered data) Node C: Authorized secure node (AES + HMAC + anomaly detection + cloud blocking) Flask-based dashboards for real-time visualization ThingSpeak cloud interface for remote validation Attack types tested: tampering, spoofing, replay, jamming, and drop attacks
MATLAB Large-Scale IoV Simulation (100 Vehicles) (MATLAB R2024a)	Benchmark communication performance across IoV technologies and environments.	100 virtual vehicles simulated in a cross-road mobility model Technologies: 5G, LoRaWAN, and LoRa P2P Environments: urban, rural, and hill station Metrics: PDR, latency, energy usage, attack vs. mitigation rate Attack types: replay, spoof, tamper, jamming, drop Real sensor data from hardware testbed used as baseline input

**Table 3:** Performance comparison across communication technologies and environments.

Technology	5G	LoRaWAN	LoRa P2P
Cross-Road	93.5	90.1	**96.9**
Urban	96.4	87.1	**96.1**
Hill	80.9	94.2	**99.5**
Rural	88.7	92.1	**99.5**
Avg. PDR (%)	89.9	90.9	**98**
Avg. Mitigation (%)	20.2	28.8	**90.4**
Energy/Tx (J)	0.0032	0.0025	**0.0018**
Remarks	High bandwidth, NLoS sensitive	Reliable, gateway dependent	Secure, efficient, most stable

*Note:* Bold values indicate the best (optimal) performance among the compared technologies for the given metric/environment.

**Table 4:** Security feature comparison: conventional vs. proposed secure TPMS framework.

Security Aspect	Conventional TPMS/IoV	Proposed Secure Framework
Communication Channel	Plain RF broadcast, unencrypted.	AES-128 CBC encryption with HMAC integrity tags.
Data Integrity	No validation or signature.	HMAC-SHA256 prevents undetected modification.
Authentication	Static IDs are easily cloned.	Unique AES key pairs ensure node authenticity.
Privacy	Fixed IDs allow tracking.	Weekly pseudonymous UID regeneration.
Replay Resistance	Accepts duplicate packets.	Timestamp and nonce-based rejection.
Cloud Security	HTTP upload (plaintext).	HTTPS with verified data and attack log.
Physical Access Control	Open ECU ports.	USB-based hardware authorisation.
Attack Resistance	Vulnerable to jamming/spoofing.	Mitigation > 90% across all simulated attacks.
Auditability	No logs.	Node C_T maintains secure tamper logs.
Energy Efficiency	High power (cellular).	1.8 mJ per transmission—most efficient.

## Data Availability

The data presented in this study are available on request from the corresponding author.

## Abbreviations

The following abbreviations are used in this manuscript:

ACU	Autonomous Control Unit
ADC	Analog-to-Digital Converter
AES	Advanced Encryption Standard
CAN	Controller Area Network
CPS	Cyber-Physical System
ECU	Electronic Control Unit
HMAC	Hash-Based Message Authentication Code
IoV	Internet of Vehicles
LPWAN	Low-Power Wide-Area Network
LoRa	Long Range
LoRaWAN	Long Range Wide Area Network
P2P	Peer-to-Peer
PDR	Packet Delivery Ratio
RF	Radio Frequency
TPMS	Tire Pressure Monitoring System
TX	Transmitter
RX	Receiver
